# MOTCS: A Cancer Subtype Classification and Key Biomarker Recognition Model Based on Multi-Omics Data Integration of Transformer

**DOI:** 10.3390/ijms27104237

**Published:** 2026-05-10

**Authors:** Ping Meng, Guohua Wang, Tianjiao Zhang

**Affiliations:** 1 Faculty of Computing, Harbin Institute of Technology, Harbin 150001, China; 19b903053@stu.hit.edu.cn; 2 School of Computer Science and Artificial Intelligence, Northeast Forestry University, Harbin 150040, China

**Keywords:** cancer subtype, multi-omics, transformer, driver gene

## Abstract

Cancer, as a complex disease with high heterogeneity, is gradually shifting from standardization to individualization. Current research indicates that integrating multi-omics data, including genome, transcriptome, and epigenome, can more comprehensively reveal the molecular features of tumors, thereby providing a more reliable basis for subtype classification. It is worth noting that driver genes, as key factors directly involved in tumorigenesis and development, when combined with multi-omics data, improve model interpretability. We proposed a cancer subtype classification model named MOTCS, which consists of four key steps: (1) The extraction of driver gene features from existing databases and the execution of preliminary feature selection for non-driver genes in different omics data; (2) the use of the transformer encoder to conduct feature representation learning for non-driver gene features and driver gene features, respectively; (3) the non-driver gene feature embeddings and driver gene feature embeddings of each omics learned are concatenated and input into the MLP classifier to obtain the classification probabilities of each subtype. (4) By integrating the above features through view association discovery networks, cross-omics feature representations are learned so as to achieve effective classification. The results show that MOTCS outperformed existing methods across all four datasets and that the introduction of driver genes will enhance the classification performance of MOTCS. MOTCS achieved precise classification of cancer subtypes by mining the feature embeddings of driver genes and non-driver gene features.

## 1. Introduction

Worldwide, cancer is a serious disease. Worldwide, there is an increase in cancer incidence and prevalence each year [1,2]. According to the GLOBOCAN [3] project, there will be 14.1 million more cancer patients worldwide in 2021, accounting for approximately 14.6% of deaths. Cancer has a high fatality rate, so it is critical to receive an early diagnosis and detection [4]. Several studies have shown that the classification of tumor types and subtypes based solely on morphological features has serious limitations, such as challenges in distinguishing cancer types, and may lead to strong biases in the identification of tumors by experts [5,6]. This will result in some cancers that are diagnosed late or that are not diagnosed until they are discovered at an advanced stage [7].

In the last few years, because of the advancements in NGS, genomic data about cancer have rapidly accumulated. As an example, the TCGA [8] database includes vast cancer genomics data. Numerous studies have found that the advancement of precision medicine benefits from integrating different omics data from the same patient. Multi-omics data can reveal patient characteristics from different perspectives. Therefore, we can study the subtype classification and molecular clinical characteristics of cancer from the perspective of multi-omics data.

However, omics data has many features, a phenomenon known as the “curse of dimensionality”. Based on this, most research is still confined to single-omics data. However, current studies have integrated multi-omics to classify cancer subtypes. For instance, MODILM proposed a novel model which leverages graph attention networks and a multilayer perceptron to integrate multi-omics data, leading to a marked improvement in classification accuracy for complex disease [9]. By employing autoencoders, Rakshit et al. successfully classified cancer subtypes [10]. MOCSC is a model based on stacked sparse denoising autoencoders and single-layer neural networks [11]. However, these models are like a black box and often lack a certain degree of interpretability. Therefore, the model developed by Wang et al., based on GCN, can undertake cancer subtype classification tasks and identify key molecular markers [12]. Wang classified breast cancer subtypes and the identification of biomarkers by fusing transformer and graph convolutional networks [13].

Although cancer driver genes are essential for cancer, their value remains underexplored in current deep learning models. Integrating such gene systems with clear biological significance into the model framework is expected to significantly enhance its predictive performance and biological interpretability. As far as we know, there is currently no research that combines cancer driver genes with deep learning models. Accordingly, we have developed a novel classification model, MOTCS, which integrates multi-omics data centered on the incorporation of driver genes. The core idea of this model is to learn the deep feature representations of the driving features of each single omics and the pre-screened non-driving features through the transformer model and classify them using MLP. The VCDN aims to enable precise cancer subtype classification by integrating complementary information from multi-omics.

## 2. Results

### 2.1. Performance Comparison of MOTCS with Other Methods

In this section, we compared MOTCS with five methods. This includes three baseline models and two cutting-edge models for cancer classification. The baseline methods contain SVM, KNN, and RF. The most advanced models developed based on cancer classification include XOmiVAE [14], and MOGONET [12]. XOmiVAE is an interpretable model that leverages the variational autoencoders, and it is designed to quantify gene contributions to cancer and perform cancer subtype classification. MOGONET uses multi-omics data and a GCN model to classify cancer subtype and identify biomarkers. By jointly learning each omics features and cross-omics associations, it can be effectively applied to classification tasks. Table 1, Table 2, Table 3 and Table 4 respectively present the model comparison results of the BRCA, COAD, PRAD and KIPAN datasets. We used the t-test to compare MOTCS against the other methods, with the significant level set as *p* < 0.05. The experimental results show that MOTCS performs best on all datasets, with the average accuracies of BRCA, COAD, PRAD and KIPAN being 89.7%, 89.5%, 89.1% and 97.3%, respectively. Compared with the second-ranked SVC and RF, the accuracy rates have increased by 4.5%, 1.9%, 2.6%, and 1.5%, respectively. The significance test results show that, on the BRCA, COAD, and PRAD datasets, MOTCS significantly outperforms most methods (*p* < 0.05). However, on the KIPAN dataset, no significant difference was observed between MOTCS and most methods (*p* > 0.05). The above results indicate that MOTCS has outstanding performance in cancer subtype classification. In addition, we used an independent external cohort to validate model performance (Table S1).

### 2.2. Performance Comparison of MOTCS in Multi-Omics and Single-Omics

**Figure 1:**
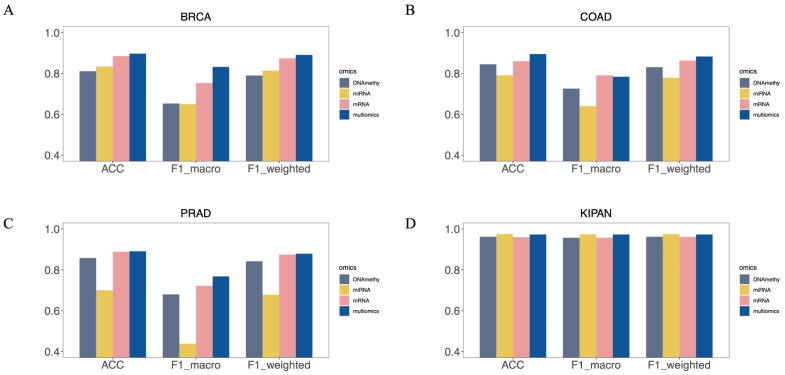
Comparison of multi-omics and single-omics data performance. (**A**) BRCA, (**B**) COAD, (**C**) PRAD (**D**) KIPAN.

Here, we evaluated different omics data for subtype classification. We constructed the MOTCS classification model for each dataset using mRNA, miRNA, and DNA methylation separately. The results show that, on each dataset, the performance achieve when jointly using multi-omics is superior to that when using single omics (Figure 1). For single-omics data, mRNA performed best on four datasets, while DNA methylation and miRNA performed differently on various datasets. The above results indicate that multi-omics data contain more comprehensive features, thereby improving classification performance.

### 2.3. The Influence of Adding Driver Genes on the Classification Performance of MOTCS

**Figure 2:**
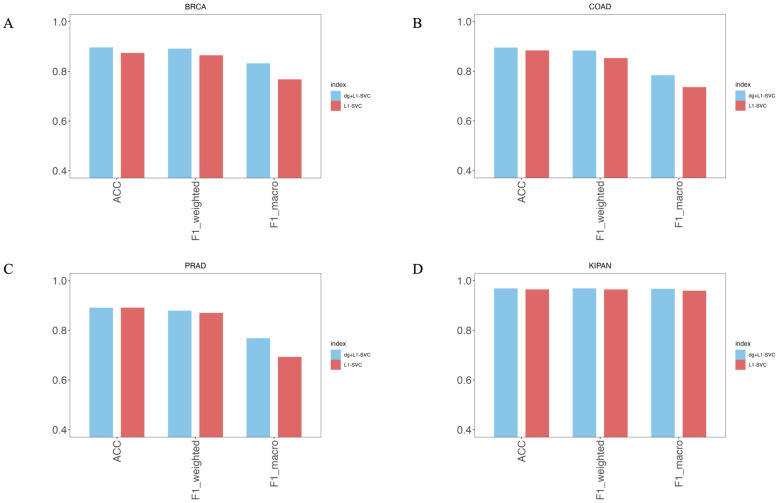
Bar chart comparing the performance of the MOTCS_dg+L1-SVC model and the MOTCS_L1-SVC model. (**A**) BRCA, (**B**) COAD, (**C**) PRAD (**D**) KIPAN.

One of the differences between the MOTCS model we constructed and other methods is that driver genes were added when building the MOTCS model. Driver genes are a class of important genes related to the development and advancement of cancers, whereas most of other omics features do not have a core pathogenic role. In addition, the number of driver genes is limited and their biological functions are clear, while most other omics features are background noise. If driver genes are mixed with other features, this will lead to the features of the driver gene being influenced and diluted by the background features. Therefore, in this study, the driver genes are treated separately, which can retain the useful information of the driver genes and improve model performance and interpretability. We benchmarked the model incorporating driver genes (MOTCS_dg+L1-SVC) against the baseline using only L1-SVC features (MOTCS_L1-SVC) to evaluate their impact to subtype classification, with results visualized in a bar chart. For the BRCA (Figure 2A), COAD (Figure 2B), and KIPAN (Figure 2D) datasets, the accuracy and F1 score of MOTCS_dg+L1-SVC are both higher than those of MOTCS_L1-SVC. For the PRAD (Figure 2C) dataset, although the accuracy of MOTCS_dg+L1-SVC is the same as that of MOTCS_L1-SVC, a higher F1 score is observed for MOTCS_dg+L1-SVC when compared with MOTCS_L1-SVC. The above results indicate that incorporating driver gene information can enhance the model’s performance.

### 2.4. The Impact of Key Parameters on the Model

**Figure 3:**
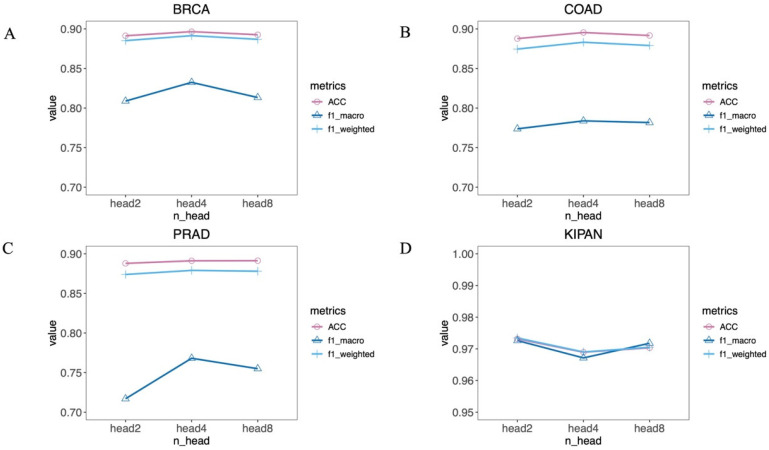
Comparison of MOTCS performance with different numbers of attention heads. (**A**) BRCA, (**B**) COAD, (**C**) PRAD, (**D**) KIPAN.

We then assessed how key parameters influence the model’s performance, with a focus on the number of attention heads and the dropout rate. The number of attention heads in the multi-head attention mechanism is a key parameter. More attention heads strengthen the model’s feature extraction capability, but this will lead to an increase in computational complexity. On the contrary, although fewer attention heads can improve computational efficiency, this may ignore the high-order nonlinear relationships among features. To explore the optimal configuration on different datasets, we constructed MOTCS models with different attention head counts across datasets for comparison. Figure 3 shows the performance comparison results of MOTCS for each dataset under different numbers of attention heads. The findings show that the optimal classification performance of MOTCS on the BRCA, COAD, PRAD, and KIPAN datasets is achieved with 4, 4, 4, and 2 attention heads, respectively.

**Figure 4:**
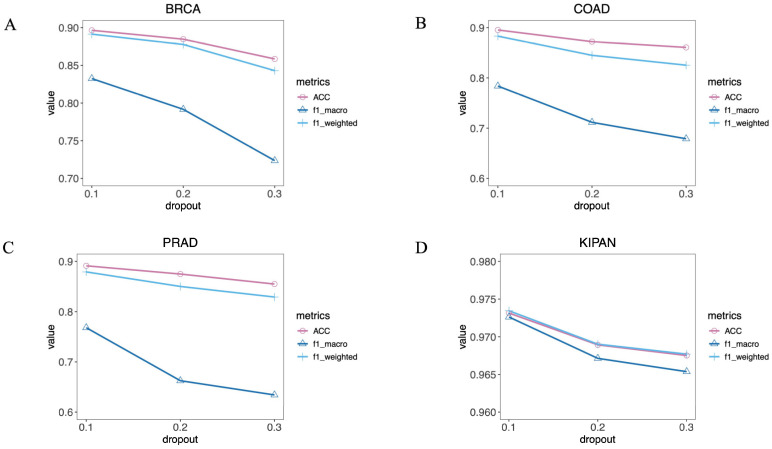
Comparison of MOTCS performance with different dropout rates. (**A**) BRCA, (**B**) COAD, (**C**) PRAD, (**D**) KIPAN.

Regarding dropout, a higher dropout rate introduces stronger random perturbations, which may lead to model underfitting, while a lower dropout rate reduces the regularization effect, thereby diminishing the generalization stability across datasets. Similarly, we constructed MOTCS models with varying dropout rates for each dataset to compare their performance. Figure 4 presents the comparative results of MOTCS performance across different datasets under varying dropout rates. The results indicate that, when dropout is set to 0.1, the BRCA, COAD, PRAD, and KIPAN datasets all achieve their optimal classification performance.

### 2.5. Identifying Important Biomarkers with MOTCS

The identification of key biomarkers is of great significance for models to explain and understand cancer occurrence and development. In this study, to conduct a feature importance analysis, we assigned each feature to 0 and calculated the model’s F1 score after a certain feature was assigned to 0. By comparing the model’s F1 score before and after a feature is set to 0, we can evaluate the feature’s importance. The greater the difference, the more important the feature is. The feature importance calculation was repeated five times. We summed up the differences of the five F1 scores and sorted them in descending order. We extracted the top 30 features of each omics and recorded them as important biomarkers.

For the classification of BRCA subtypes, the biomarkers for each omics are shown in Table 5. By conducting GO enrichment analysis on mRNA features, we identified GO terms that may be associated with BRCA, including the ERBB signaling pathway (GO: 0038127) term. ERBB receptor tyrosine kinases are key drivers in various cancers [15]. The invasiveness of breast cancer increases with ERBB receptors and their ligands [16]. ERBB2, also known as HER2, has prognostic value based on its overexpression level and is a therapeutic target for BRCA [17]. As shown in Figure S1, ERBB2 was significantly higher in the HER2 subtype than in other subtypes (*p* < 0.0001). Some chemotherapy drugs are widely used to treat breast cancer [18]. Among these, the standard treatment for HER2+ subtype BRCA is trastuzumab, which is given based on ERBB2 expression, while for Luminal B subtype breast cancer patients, endocrine therapy and the combination of trastuzumab and chemotherapy are usually adopted [19]. We found that ERBB2 is a marker for identifying the HER2 subtype, which indicates that patients classified into this subtype by our model may benefit from HER2-targeted therapy. Therefore, our model has potential clinical value in the stratification and targeted therapy of breast cancer patients. ERBB2 is also a target for fluorescence molecular imaging. Previous studies have successfully developed antibody–drug conjugates (ADCs) based on HER2 antibodies. This ADC simultaneously contains the near-infrared fluorescent dye Cy5 for fluorescence imaging and the chemotherapy drug SN38 for targeted therapy, and its anti-tumor activity has been confirmed in patients with HER2-positive breast cancer [20]. Additionally, as a SOX family gene, SOX11 is key to cell differentiation and to determining cell fate [21]. SOX11 demonstrated tumor-specific expression, being upregulated in breast cancer but not in normal breast epithelium. Research indicates that high SOX11 expression contributes to poor prognosis of breast cancer [22]. SOX11 predicts poor survival outcomes in breast cancer [23]. SPC25 is an active protein involved in the filament–microtubule interaction and exhibits high expression in lung, prostate, and breast cancer. SPC25 may influence the occurrence and development of breast cancer by regulating pathways [24]. We conducted KEGG pathway analysis on the top 30 mRNA features of BRCA. The results indicated that these features were notably enriched in multiple classical cancer pathways, including the ErbB signaling pathway, EGFR tyrosine kinase inhibitor resistance, the PI3K-Akt signaling pathway, and platinum drug resistance (FDR < 0.05). It is worth noting that the ErbB signaling pathway was also significantly enriched in the GO enrichment analysis, and this result verified the reliability of our pathway analysis. Previous studies have shown that EGFR tyrosine kinase inhibitors (EGFR-TKI) competitively block ATP binding to EGFR, thereby inhibiting EGFR activation [25]. EGFR phosphorylation activates the downstream PI3K/AKT pathways, which regulate cell proliferation and survival. The activation of this pathway is the main cause of therapeutic drug resistance [26] and is also of great value for the prognosis of BRCA patients [27]. Platinum-based anticancer drugs kill cancer cells by inducing DNA damage and are a standard treatment for solid tumors [28]. By conducting GO enrichment analysis on DNA methylation features, some GO terms may drive breast cancer progression. For example, negative regulation of the intracellular steroid hormone receptor signaling pathway (GO: 0033144). Steroid hormone secretion, signal transduction, and dysfunction can cause various types of cancer [29]. Estrogen receptor α (ER-α) regulates cell cyclin-related genes, thereby promoting the growth of breast epithelium. Moreover, when hormone secretion is abnormal, it activates the ER pathway, thereby stimulating cell proliferation and elevating cancer risk [29]. There is also some evidence that miRNA features have been associated with BRCA. For instance, patients with breast cancer exhibiting high miR-93-5p expression tend to have a poor prognosis [30], as miR-93-5p is a regulator of tumorigenesis and immunity through direct targeting of the PD-L1/CCND1 pathway [31]. Meanwhile, miR-10b-5p expression is linked to metastasis of BRCA and it drives metastasis through the HOXD10/RHOC axis [32].

For the COAD subtype classification model, the biomarkers for each of the omics are shown in Table 6. By conducting GO enrichment analysis on mRNA characteristics, we found that regulation of epithelial to mesenchymal transition (GO: 0010717) may be related to COAD. Epithelial–mesenchymal transition (EMT) is important to human development. EMT promotes migration in epithelial cancer cells, which in turn facilitates invasion and metastasis [33]. Previous studies have analyzed the markers involved in the EMT-related pathways and discovered their expression patterns in COAD patients [34]. Additionally, overexpression of AGR2 is correlated with poorer OS in cancer patients [35]. Exogenous AGR2 promotes an aggressive phenotype in COAD [36]. KEGG pathway analysis was conducted on the top 30 mRNA features of COAD. Some pathways are significantly enriched, including colorectal cancer, PD-L1 expression and PD-1 checkpoint pathway in cancer, EGFR tyrosine kinase inhibitor resistance, TGF- β signaling pathway and the FoxO signaling pathway (FDR < 0.05). Among these, the enrichment of the colorectal cancer pathway directly verified that these features were associated with COAD. PD-1/PD-L1 immunotherapy is a primary cancer treatment, and response depends on PD-L1 expression [37]. Regarding miRNA features, existing research indicates that miR-155-5p shows an up-regulated expression pattern in COAD tissues. Overexpression of miR-155-5p stimulates COAD cell proliferation and enhances their invasion and metastasis abilities [38].

For the PRAD subtype classification model, the biomarkers for each of the omics are shown in Table 7. By performing GO enrichment analysis on the mRNA characteristics of PRAD, we found that some GO terms may be related to PRAD, including androgen receptor signaling pathway (GO: 0030521). Androgen receptor (AR) is a ligand-induced transcription factor [39], which regulates the transcription process of genes by binding to the androgen response elements of target genes [40]. In PRAD, the mRNA and protein expression levels of AR were significantly increased [41]. AR can form complexes with heat shock proteins (HSPs) to recruit integrin β1 and activate Rac1 and FAK, thereby driving cell migration and influencing the progression and metastasis of PRAD [40]. For DNA methylation features, PARPs are a class of multifunctional protein translation-modifying enzymes. PARP6 is primarily distributed in well-differentiated adenocarcinoma cells [42]. As shown in Figure S2, PARP6 methylation was significantly lower in the SPOP than others (*p* < 0.001). Existing studies have shown that hypermethylation of PARP6 is significantly associated with disease-specific mortality in PRAD [43]. Our research found that PARP6 in the SPOP subtype shows hypomethylation, and that the SPOP mutant PRAD has a better clinical prognosis [44], which is completely consistent with the lower mortality rate indicated by PARP6 hypomethylation. KEGG pathway analysis was performed on the top 30 mRNA features of PRAD, and no significantly enriched pathways were detected. Pathways with higher enrichment scores include notch signaling pathways and transcriptional misregulation in cancer. Regarding miRNA features, studies have found that miR-193a-5p is abnormally expressed in PRAD. miR-193-5p also binds to circAMOTL1L to inhibit the protocadherin gene cluster. Knockdown of miR-193-5p leads to dysregulation of the circAMOTL1L–miR-193-5p–PCDHA8 regulatory pathway, thereby promoting the development of PRAD [45].

### 2.6. Analysis of the Tumor Microenvironment of Cancer Subtype

**Figure 5:**
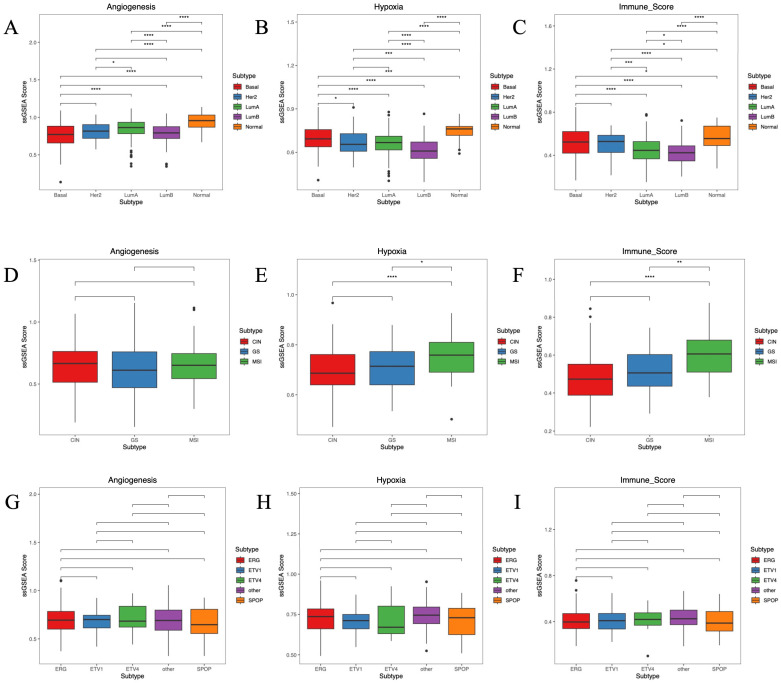
Tumor microenvironment of different cancer subtypes. (**A**) The angiogenesis enrichment score of the BRCA subtype. (**B**) The hypoxia enrichment score of the BRCA subtype. (**C**) The immune enrichment score of the BRCA subtype. (**D**) The angiogenesis enrichment score of the COAD subtype. (**E**) The hypoxia enrichment score of the COAD subtype. (**F**) The immune enrichment score of the COAD subtype. (**G**) The angiogenesis enrichment score of the PRAD subtype. (**H**) The hypoxia enrichment score of the PRAD subtype. (**I**) The immune enrichment score of the PRAD subtype. * *p* < 0.05, ** *p* < 0.01, *** *p* < 0.001, **** *p* < 0.0001.

To compare tumor microenvironment among cancer subtypes, we used GSVA to calculate the enrichment scores of each sample in angiogenesis, hypoxia, and immunity score. BRCA subtypes were significantly different (*p* < 0.001) in angiogenesis, hypoxia, and the immune score (Figure 5A–C). For COAD, the MSI subtype differed significantly from CIN (*p* < 0.0001) and GS (*p* < 0.05) subtypes in hypoxia (Figure 5E). For immune score, significant differences were found between the CIN and MSI subtypes (*p* < 0.0001) and between the GS and MSI subtypes (*p* < 0.01) (Figure 5F), but no significant differences were found in angiogenesis (Figure 5D). For PRAD, there were no significant differences among the three TMEs in each subtype (Figure 5G–I).

## 3. Discussion

This study proposed a cancer subtype classification model named MOTCS. This model innovatively combines transformer, VCDN and driver genes and is applied to the task of cancer subtype classification for the first time. The multi-head attention mechanism of transformer can learn the intrinsic correlations between features without relying on any prior knowledge, making it more suitable for high-dimensional multi-omics data. VCDN can further explore the potential correlations among different omics data types in the high-dimensional label space and achieve the integration of prediction results from various omics data. The addition of driver genes can further enhance the classification performance of the model. By comparing with other existing multi-omics models, we verified the superiority of this combined strategy in the task of cancer subtype classification.

Comparing the model’s performance with single-omics, we found that mRNA expression data outperformed single-omics across all four datasets. The performance of DNA methylation and miRNA expression data varies across datasets. For the BRCA dataset, miRNA performed better. The DNA methylation data performed better in the COAD and PRAD datasets. The classification performance of multi-omics data is the best, fully confirming the necessity of combining coding genes, non-coding genes and epigenetic features.

Our study improved cancer subtype classification by integrating multi-omics data, but this method has certain limitations in clinical application. Under conventional clinical conditions, DNA methylation sequencing encounters problems such as complex experimental procedures, high sample quality requirements, and high sequencing costs, making it difficult to be widely applied. To address this challenge, our model framework supports classification using only mRNA data and performs nearly as well as multi-omics. Future work will focus on simplifying model structure, reducing dependency on DNA methylation data, and enhancing clinical applicability.

Given the high cost and difficulty of obtaining multi-omics data, as well as the fluctuations in the ranking of feature importance across different datasets, it is challenging to determine a minimum biomarker panel. Therefore, we have provided the top 30 features of each omics for researchers to choose flexibly. In the future, we will further screen and validate the minimum marker panel in larger independent clinical cohorts.

In this study, we evaluated feature importance by removing features one at a time and measuring the decrease in F1 score. This method is widely used in multi-omics biomarker studies. However, it may be limited by correlation among features. Despite this, enrichment analysis showed that the identified biomarkers have clear biological functions. Future studies could adopt methods such as SHAP for cross-validation to obtain more robust assessments of feature importance.

In addition, this study built a cancer subtype classifier, focusing on the molecular differences among subtypes. The relationship between each subtype and the expression of photodynamic therapy (PDT) related target proteins has not yet been further investigated. The effective binding of photosensitizers involves complex biological processes, and different photosensitizers target different proteins. Currently, we lack the systematic data to construct predictive models. In the future, we hope to further expand the research in this direction by combining relevant data.

Although the MOTCS model has achieved good results, there is still room for further performance improvement. Future research will aim to enhance the model’s performance from the following aspects: Firstly, adding multiple omics data types. The current model is mainly based on transcriptomic and epigenomic data. In the future, we plan to incorporate proteomic and metabolomic information, etc., to deeply explore the potential associations among multiple omics levels. Secondly, optimize the feature weights. At present, the model’s treatment of driver genes and non-driver genes has not fully reflected the differences in their biological significance. We plan to design a learnable adaptive weighting mechanism to assign different weights to driver genes and non-driver genes during the model training process, in order to enhance the classification performance of the model.

## 4. Materials and Methods

**Figure 6:**
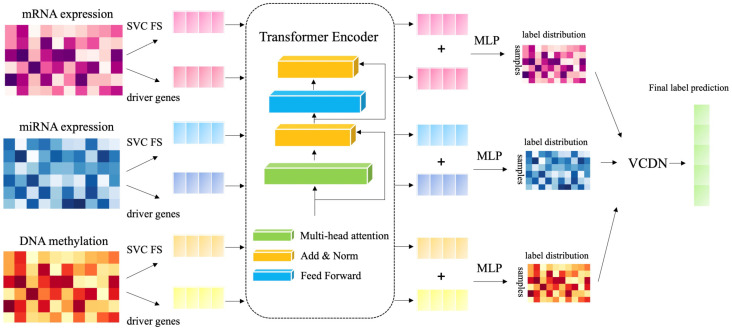
The overall framework of this method.

Here, we mainly introduce the overall workflow of MOTCS (Figure 6). MOTCS mainly consists of three parts: (1) Preprocessing. Each omics dataset was preprocessed and subjected to feature selection to retain the features most closely related to the classification task. (2) Transformer model. The transformer model is applied to the retained non-driver features and driver gene of each omics data. The model first leverages its self-attention mechanisms to deeply mine feature associations within the omics data. Based on this, it extracts a biologically meaningful feature representation, which is finally used by MLP for preliminary classification. (3) Multi-omics integration based on VCDN. After obtaining the prediction probabilities of each omics data, VCDN integrates those results for final prediction.

### 4.1. Data Acquisition

In this work, we downloaded the multi-omics data with the R package “TCGAbiolinks” [46]. The dataset encompasses six cancer types (BRCA, COAD, PRAD, KICH, KIRC, and KIRP) and includes mRNA, miRNA, and DNA methylation data. For each cancer type, we retained only samples with complete multi-omics profiles. Finally, we obtained a total of 2033 samples, including 6 types of cancer (Table 8). BRCA subtypes include Normal, HER2, Basal-like, Luminal A, and Luminal B. The COAD dataset comprises three subtypes, namely GS, CIN, and MSI [47]. The PRAD dataset includes five subtypes, namely ERG, ETV1, ETV4, SPOP, and other [48]. KIPAN is used for the classification of renal cancer, including three types of renal cancer, namely KICH, KIRC, and KIRP, and normal samples.

The list of driver genes used in this study is derived from two databases and the list of coding driver genes is from the NCG [49] database. Rather than coding driver genes, miRNAs closely related to tumorigenesis were obtained from the OncomiR [50] database and systematically defined as non-coding driver genes in this study.

### 4.2. Data Preprocessing

Omics data have many features, something that is often called the “curse of dimensionality”. However, only a few features are related to clinical symptoms or features. Therefore, to overcome the “curse of dimensionality” and mitigate overfitting. We need to perform feature selection before performing downstream analysis. First, for mRNA and miRNA, we removed features (mRNA/miRNA) that are 0 in more than 25% of samples. For DNA methylation data, we filtered out CpGids that are not expressed in more than 25% of the samples and used CHAMP to filter out CpGids and SNPs on the XY chromosomes. Then, we imputed the missing values using the imput.knn function. Finally, the mean methylation value of the CpGids at 1500 bp upstream of the TSS locus of the gene was taken and recorded as the methylation level for that gene.

Next, based on the known list of coding and non-coding driver genes, we systematically classified features into two major categories: driver features and non-driver features. Under this framework, we conducted feature selection for the non-driver features of each omics using L1-regularized linear support vector classification (L1-SVC). L1 regularization zeros out irrelevant features, enabling effective feature selection. This method identifies key features related to cancer subtype from high-dimensional omics data, eliminates the influence of noise features on the model, and enhances model interpretability. We set the penalty parameter C of L1-SVC to 0.1 to obtain a sparser feature set and maintain consistent parameter configuration across all datasets. To validate this parameter, we compared model performance with C = 0.5 and C = 1, C = 0.1 achieves the best performance in all datasets (Figure S3). Feature stability was assessed by calculating the pairwise overlap proportion of selected features across folds in 5-fold cross-validation.

As the driver gene features themselves usually have clear biological significance and a relatively limited number, we retained all their information to ensure the integrity of the driver genes.

### 4.3. Transformer Model

In the MOTCS framework, considering the differences in multi-omics data and the limitations of the existing PPI network, we adopt the transformer model to conduct deep feature learning on each omics data. The multi-head self-attention mechanism captures implicit feature correlations without relying on predefined graph priors. This model respectively learns the driver and non-driver features of each omics to mine deep feature representations. Transformer is characterized by its attention mechanisms and has shown outstanding efficacy in natural language processing (NLP) [51]. Transformer has an encoder and a decoder. Among these, the transformer encoder module mainly conducts deep context modeling and learning representations from the input data. The encoder comprises multiple identical layers, each of which includes both a self-attention layer and a feed-forward network layer.

In the original transformer model, position encoding is a key design and is mainly used to make up for the permutation invariance inherent in the self-attention mechanism, itself achieved by introducing position information to distinguish the sequential relationship of different elements in the sequence. However, in our classification task, there is no sequential relationship among the input features, and their inherent order is irrelevant to the model’s results. Therefore, in order to simplify the model structure and fit the task characteristics, we proactively omitted the position encoding module in the model design, thereby focusing more on the semantic information extraction of the features themselves and avoiding the introduction of unnecessary position biases.

Therefore, each omics dataset is fed into the transformer as a feature matrix, denoted as X$\in R^{M\times N}$ where M and N are the sample size and feature dimension, respectively. The input matrix X$\in R^{M\times N}$ will first pass through a linear embedding layer and obtain the embedding matrices E$\in R^{M\times N\times D}$, where D is the high-dimensional feature space after linear mapping. Let query (Q), key (K), and value (V) be the query, key, and value vector matrix. Therefore, to compute self-attention, we used the following formula:

(1) $$SelfAttention(Q,K,V)=softmax(\frac{QK^{T}}{\sqrt{d_{k}}})V\;Q=E\times W^{Q}\;K=E\times W^{K}\;V=E\times W^{V}$$

where $W^{Q}$, $W^{K}$, and $W^{V}$ represent the learnable weight matrix.

Then, extend the self-attention to multi-head attention, and the calculation formula is as follows:

(2) $$\mathrm{MultiHead}\;(Q,\;K,\;V)\;=\;\mathrm{Concat}({head}_{1},\ldots ,{head}_{h})W^{0}$$

Then, the output matrix of the multi-head attention will be input into a fully connected layer. Ultimately, we will obtain the feature representation of each sample.

(3) $$\mathrm{FFN}(X)=\max\;(0,\;XW_{1}+b_{1})W_{2}+b_{2}$$

For each omics view $V_{m}\in$ v(where m = 1, 2, 3 represents mRNA, miRNA, and DNA methylation, respectively), we first utilize the transformer encoder to extract deep feature representation matrices $Z_{m}^{dg}$ and $Z_{m}^{svc}$ from the driver feature matrix $X_{m}^{dg}$ and the SVC-L1 feature matrix $X_{m}^{svc}$, respectively. Subsequently, the two feature representation matrices of the same view are horizontally concatenated to obtain the global feature representation matrix $Z_{m}$ for that view, i.e., $Z_{m}$ = cat ($Z_{m}^{dg}$,$Z_{m}^{svc}$). Then, we used the MLP classifier to generate the predicted probabilities for each omics view, which can be expressed as follows:

(4) $${\hat{Y}}_{\;\;\;tr}^{\left(m\right)}=\;{MLP}_{m}(Z_{tr}^{m})$$

where ${\hat{Y}}_{\;\;\;tr}^{\left(m\right)}$ ∈ $R^{n\times c}$. We denoted the j-th row of ${\hat{Y}}_{\;\;\;tr}^{\left(m\right)}$ as ${\hat{Y}}_{\;\;\;\;\;j}^{\left(m\right)},$ which represents the predicted probability for the label of the j-th sample from m-th omics type. Thus, the loss function for the MLP is as follows:

(5) $$L_{MLP,m}=\;-\frac{1}{n}\sum_{j=1}^{n}\sum_{k=1}^{c}y_{j,k}^{m}\cdot \log\;({\hat{y}}_{\;\;j,k}^{\left(m\right)}+\epsilon )$$

where $L_{MLP,m}$ is the cross-entropy loss function, n represents the sample quantity, and c represents the sample category.

### 4.4. VCDN Cross-View Fusion and Final Classification

In previous studies, it was generally found that multi-omics features were directly spliced together. However, this is prone to losing key discriminative information. Therefore, we used the VCDN to fuse the label prediction probabilities from each omics, which can integrate multi-omics information and enhance classification accuracy. We designated the views as follows: V1 (mRNA), V2 (miRNA), and V3 (DNA methylation). Denote the predicted probability distribution for the j-th training sample from view m as ${\hat{y}}_{\;\;\;\;\;j}^{\left(m\right)}$ ∈ $R^{c}$ (m = 1, 2, 3, representing three types of omics, and c is the cancer subtypes number), and let ${\hat{y}}_{\;\;\;j,c}^{\left(m\right)}$ be the probability of this sample being assigned to class c. We constructed a cross-omics tensor to capture the cross-view tensor, where each element represents the predicted probability that the j-th sample belongs to a specific class of the three views, as formulated below:

(6) $$C_{j,c_{1},c_{2},c_{3}}=\;{\hat{y}}_{j,c_{1}}^{\left(1\right)}\cdot {\hat{y}}_{j,c_{2}}^{\left(2\right)}\cdot {\hat{y}}_{j,c_{3}}^{\left(3\right)}$$

Then, reshape tensor $C_{j}$ into tensor a vector, where the combined probability is passed to a fully connected network for which the output is c. The VCDN loss function is as follows:

(7) $$L_{VCDN}=\frac{1}{n}\sum_{j=1}^{n}L_{CE}(\mathrm{VCDN}\;({\hat{y}}_{\;\;\;\;j}^{\left(1\right)},{\hat{y}}_{\;\;\;\;j}^{\left(2\right)},{\hat{y}}_{\;\;\;\;j}^{\left(3\right)}),y_{j})$$

Finally, the total loss function of MOTCS is as follows:

(8) $$L\;=\;\sum_{m=1}^{3}MLP+\;L_{VCDN}$$

### 4.5. Experimental Settings

We implemented the MOTCS model based on Python 3.9 and PyTorch 2.3.1 framework. All four datasets used the same optimizer, Adam, with the epoch set to 100, an early stop strategy 50, and random seed to 42. Through parameter optimization, the dropout rate was set to 0.1, the learning rate to 1 × 10^−5^, and the batch size to 128. The number of attention heads was set to 4, 4, 4, and 2 for BRCA, COAD, PRAD, and KIPAN datasets, respectively.

### 4.6. Data Partitioning and Evaluation Metrics

In this study, we used a 5-fold cross-validation method to divide the whole data to verify the model’s robustness. Specifically, we divided the whole dataset into five subsets—one for the test set and four for the training dataset. In each division, we used the training data for feature selection and for training the model, while the test data were employed to assess performance. The final results were obtained by averaging the outcomes from all five runs.

To assess the model’s performance, we select several indicators for assessment. Due to the class imbalance in the dataset, we adopt ACC, weighted F1 score (F1_weighted) and macro F1 score (F1_macro) as the main evaluation metrics. Among these, the weighted F1 score assigns a weight to each subtype based on the sample proportion, which can reflect the performance of the model in each class. The macro F1 score is calculated by taking the average of the F1 score of each subtype separately.

### 4.7. ssGSEA Analysis

We downloaded the Hallmark gene set from the MSigDB database to obtain the angiogenesis-, hypoxia-, and immune-related gene sets. The enrichment scores of each sample in angiogenesis, hypoxia, and immunity were calculated using the R package GSVA.

## 5. Conclusions

Overall, MOTCS achieves precise classification of cancer subtypes and identification of key biomarkers, using the transformer model to learn multi-omics deep embedding features of driver genes and non-driver genes, and the efficient collaborative fusion of VCDN to integrate multi-omics information.

## Figures and Tables

**Table 1:** Performance comparison for BRCA subtype classification.

	ACC	F1_Weighted	F1_Macro
MOTCS	0.897 ± 0.024	0.891 ± 0.029	0.832 ± 0.056
SVC	0.852 ± 0.029 *	0.836 ± 0.037 *	0.736 ± 0.074 *
KNN	0.788 ± 0.03 *	0.751 ± 0.038 *	0.642 ± 0.093 *
RF	0.862 ± 0.03 *	0.846 ± 0.041 *	0.734 ± 0.075 *
XOmiVAE	0.824 ± 0.045 *	0.822 ± 0.046 *	0.733 ± 0.08 *
MOGONET	0.859 ± 0.038 *	0.853 ± 0.038 *	0.76 ± 0.063 *

Note: * means MOTCS achieved significantly better performance than the baseline method.

**Table 2:** Performance comparison for COAD subtype classification.

	ACC	F1_Weighted	F1_Macro
MOTCS	0.895 ± 0.022	0.883 ± 0.023	0.784 ± 0.102
SVC	0.876 ± 0.037	0.853 ± 0.046	0.738 ± 0.09
KNN	0.798 ± 0.051 *	0.804 ± 0.052 *	0.704 ± 0.074 *
RF	0.853 ± 0.034 *	0.812 ± 0.051 *	0.659 ± 0.058 *
XOmiVAE	0.806 ± 0.04 *	0.813 ± 0.033 *	0.704 ± 0.067 *
MOGONET	0.795 ± 0.048 *	0.796 ± 0.042 *	0.698 ± 0.047

Note: * means MOTCS achieved significantly better performance than the baseline method.

**Table 3:** Performance comparison for PRAD subtype classification.

	ACC	F1_Weighted	F1_Macro
MOTCS	0.891 ± 0.042	0.879 ± 0.047	0.768 ± 0.054
SVC	0.849 ± 0.053	0.826 ± 0.054	0.639 ± 0.071 *
KNN	0.775 ± 0.075 *	0.749 ± 0.092 *	0.558 ± 0.12 *
RF	0.865 ± 0.049 *	0.846 ± 0.052 *	0.678 ± 0.086 *
XOmiVAE	0.849 ± 0.059	0.849 ± 0.057	0.727 ± 0.091
MOGONET	0.773 ± 0.103	0.783 ± 0.087	0.592 ± 0.082 *

Note: * means MOTCS achieved significantly better performance than the baseline method.

**Table 4:** Performance comparison for KIPAN subtype classification.

	ACC	F1_Weighted	F1_Macro
MOTCS	0.973 ± 0.022	0.973 ± 0.022	0.973 ± 0.034
SVC	0.955 ± 0.019	0.955 ± 0.019	0.952 ± 0.027
KNN	0.955 ± 0.022	0.955 ± 0.022	0.952 ± 0.028
RF	0.956 ± 0.022	0.956 ± 0.021	0.954 ± 0.029
XOmiVAE	0.958 ± 0.021 *	0.958 ± 0.021 *	0.954 ± 0.033 *
MOGONET	0.942 ± 0.024	0.941 ± 0.026	0.929 ± 0.046

Note: * means MOTCS achieved significantly better performance than the baseline method.

**Table 5:** Biomarkers for BRCA subtype classification.

Omics Data Type	Biomarkers
mRNA expression	**SOX11**, **RRAS2**, MPHOSPH6, **UFD1**, **AURKA**, ZNF98, CDH3, **HNRNPC**, **SPC25**, TNFAIP3, CBLB, **AKT1**, **MCL1**, **SETD2**, CENPO, **ARID1A**, **PARP4**, **PIK3CA**, FAM171A1, MICALL1, PGAP3, GK5, FSCN3, RIPPLY3, TBC1D31, **FOXA1**, **ERBB2**, SCNN1A, **KNSTRN**, MSH2
DNA methylation	MCCD1, **MUC16**, RP11-475O23-3, **PCDHB11**, IGFBP4, RP11-158I23-1, **SERPINA11**, CTD-2207O23-12, **ELTD1**, MT1E, CKS2, PAPPA2, CYBA, CHAD, **FREM1**, OR5B17, SBNO2, AOAH, C7orf62, **SPINK1**, RSAD1, ZNF358, CHPF, CDKN1B, **FOXP1**, **MDM2**, RHBG, **TP63**, USH2A, CHCHD2,
miRNA expression	**hsa-miR-93-5p**, hsa-let-7i-5p, **hsa-miR-497-5p**, hsa-miR-369-5p, **hsa-miR-671-5p**, hsa-miR-10b-5p, hsa-miR-625-5p, hsa-miR-16-5p, hsa-miR-877-5p, hsa-miR-181b-5p, **hsa-miR-769-5p**, **hsa-miR-98-5p**, hsa-let-7c-5p, hsa-miR-99a-5p, hsa-miR-146b-5p, hsa-miR-378a-5p, hsa-miR-4746-5p, hsa-miR-103a-2-5p, hsa-miR-3074-5p, hsa-miR-1271-5p, hsa-miR-320c, **hsa-let-7d-5p**, hsa-miR-2110, hsa-miR-224-5p, **hsa-miR-3651**, hsa-miR-378c, **hsa-miR-424-5p**, hsa-miR-432-5p, hsa-miR-503-5p, hsa-miR-511-5p

Bold: Features that have been reported in existing literature and verified through biology.

**Table 6:** Biomarkers for COAD subtype classification.

Omics Data Type	Biomarkers
mRNA expression	ATP5F1A, C6orf136, **FBXW7**, **MAP2K1**, **MAP7**, MOV10, **MUC4**, PRKCQ, **SIN3A**, **SMAD2**, **SMAD4**, TMEM201, **AGR2**, **FAM189A1**, GGT6, MCUB, **OXCT1**, ZMYND12, CHMP7, RPL22L1, **CNDP2**, SERPINB8, **KDM6A**, **PTEN**, SAMM50, ADAT, ANKRD39, **CDCA2**, CTDP1, CYB5D2
DNA methylation	ELL2P1, **ZFR**, SEC61A2, KRCC1, **FLYWCH1**, RNF19A, RBM12, **RB1**, **CDK19**, MLH1, FBXW7, FSD2, HTR2B, NTSR1, **SPATA12**, **TEX14**, TRIM45, **EPM2AIP1**, **AXIN2**, RP11-649E7-5, AC104066-1, ECM2, ERVMER34-1, LCLAT1, RP11-46I8-3, ZFP3, **ASXL1**, CCDC102A, CSMD3, DAO
miRNA expression	hsa-miR-1266-5p, **hsa-miR-142-5p**, hsa-miR-33b-5p, hsa-miR-744-5p, hsa-miR-3934-5p, hsa-miR-130b-5p, **hsa-miR-330-5p**, hsa-miR-375, hsa-miR-155-5p, hsa-miR-22-5p, hsa-miR-200a-5p, hsa-miR-643, hsa-miR-10b-5p, **hsa-miR-186-5p**, hsa-miR-628-5p, hsa-miR-146b-5p, hsa-miR-2110, hsa-miR-223-5p, hsa-miR-3127-5p, hsa-miR-320a, hsa-miR-582-5p, hsa-miR-592, hsa-miR-874-5p, hsa-miR-4772-5p, hsa-miR-625-5p, hsa-let-7a-5p, hsa-let-7b-5p, hsa-let-7c-5p, hsa-let-7e-5p, hsa-let-7f-5p

Bold: Features that have been reported in existing literature and verified through biology.

**Table 7:** Biomarkers for PRAD subtype classification.

Omics Data Type	Biomarkers
mRNA expression	**VSTM2L**, GOPC, RIOK2, PABPC1L2B, **TBX1**, AUTS2, AIDA, ZNF671, **CHD1**, AKIRIN2, PLIN2, **PGM3**, CDKN1B, AFG1L, IWS1, HEXA, TBCK, **ETV1**, **GHR**, **ARID1A**, JHY, NCDN, SLC16A9, **MED12**, **MLH1**, NCOR2, ARFGAP2, **GABRB3**, **HDAC1**, MLLT6
DNA methylation	PARP6, OR8K3, TNFSF12, HTR3B, MARK2, PRKCQ-AS1, BLID, C1QL3, C3orf14, TBX3, OR3A2, OR6M1, PRKCQ, ARHGDIB, TDRD1, SERINC4, **SERPINB2**, TEX37, MIOS, MUC3A, OR5K3, RP11-646J21-6, RP11-489D6-2, CPNE8, GNAS, **IDH1**, SCN11A, CYB5R4, RYR3, **KMT2D**
miRNA expression	hsa-miR-29b-2-5p, hsa-miR-561-5p, **hsa-miR-7-5p**, hsa-miR-500a-5p, hsa-miR-106b-5p, hsa-miR-191-5p, hsa-miR-96-5p, hsa-miR-624-5p, hsa-miR-193b-5p, hsa-miR-26b-5p, hsa-miR-103a-2-5p, hsa-miR-18a-5p, hsa-miR-192-5p, hsa-miR-29a-5p, hsa-miR-30c-5p, hsa-miR-504-5p, hsa-miR-708-5p, hsa-miR-3926, hsa-miR-532-5p, hsa-miR-590-5p, hsa-miR-629-5p, hsa-miR-30b-5p, hsa-miR-362-5p, hsa-miR-148b-5p, hsa-miR-196a-5p, hsa-miR-30a-5p, hsa-miR-6761-5p, hsa-miR-186-5p, hsa-miR-365a-5p

Bold: Features that have been reported in existing literature and verified through biology.

**Table 8:** The datasets used in this study.

Dataset	Categories	Number of Feature mRNA, Meth, miRNA
BRCA	Luminal A: 417, Luminal B: 139, Basal-like: 127, HER2-enriched: 46, Normal: 34	16,500, 21,093, 153
PRAD	ERG: 149, ETV1: 25, ETV4: 14, SPOP: 35, other: 80	16,662, 21,146, 357
COAD	CIN: 177, GS: 34, MSI: 47	16,153, 21,138, 399
KIPAN	KIRC: 322, KIRP: 275, KICH: 65, Normal: 47	16,307, 21,153, 349

## Data Availability

The datasets and codes are available at https://github.com/mping315/MOTCS (assessed on 8 May 2026).

## Supplementary Materials

The following supporting information can be downloaded at: https://www.mdpi.com/article/10.3390/ijms27104237/s1.
