# Supplementary material for: MOTCS: A Cancer Subtype Classification and Key Biomarker Recognition Model Based on Multi-Omics Data Integration of Transformer
Source: Int J Mol Sci. 2026 May 10;27(10):4237. doi: 10.3390/ijms27104237 (PMC13207841; doi:10.3390/ijms27104237)
Supplement: Supplementary file 1 [file ijms-27-04237-s001.zip › ijms-4216035-supplementary.pdf]

MOTCS: A cancer subtype classification and key biomarker recognition model based on multi-omics data integration of Transformer

Ping Meng [1], Guohua Wang [1*] and Tianjiao Zhang [2*]

1 Faculty of Computing, Harbin Institute of Technology, Harbin 150001, China;

2 School of Computer Science and Artificial Intelligence, Northeast Forestry University, Harbin, 150040, China;

* Correspondence: ghwang@hit.edu.cn (G.W.); tianjiaozhang@nefu.edu.cn (T.Z.)

1. Identifying important biomarkers with MOTCS

We analyzed the expression levels of ERBB2 across different subtypes in the BRCA dataset. As shown in Figure S1, the expression level of ERBB2 in the HER2 subtype is significantly higher than that in other subtypes ($p<0.0001$). This result confirms that the ranking of feature importance in our model has biological significance.

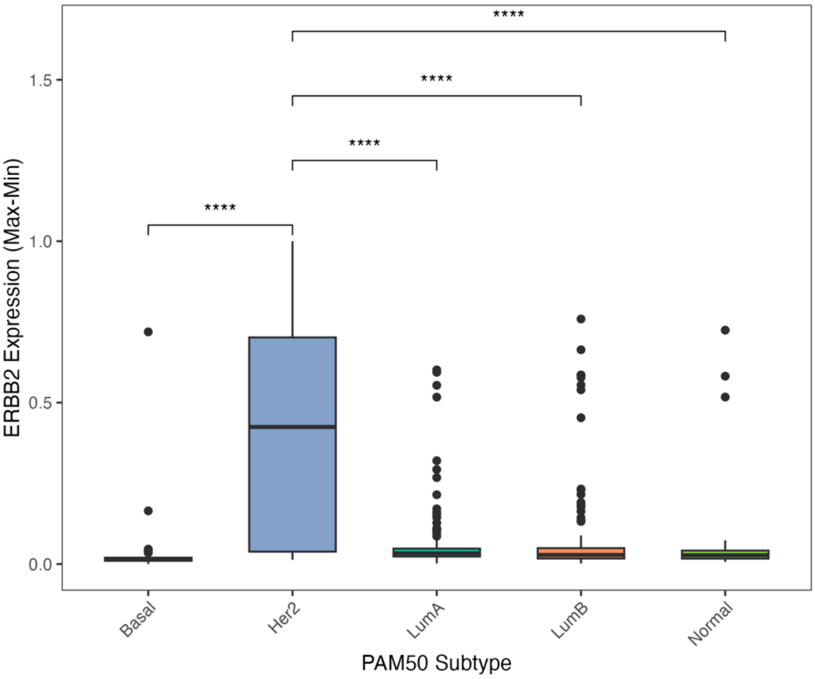

Figure S1. Bar plot showing ERBB2 expression levels across BRCA subtypes.

We analyzed the DNA methylation levels of PARP6 in each subtype of the PRAD dataset, as shown in Figure S2. The DNA methylation expression level of PARP6 in the SPOP subtype is significantly lower than that in other subtypes ($p<0.001$). This result verifies that the ranking of feature importance in our model has biological significance.

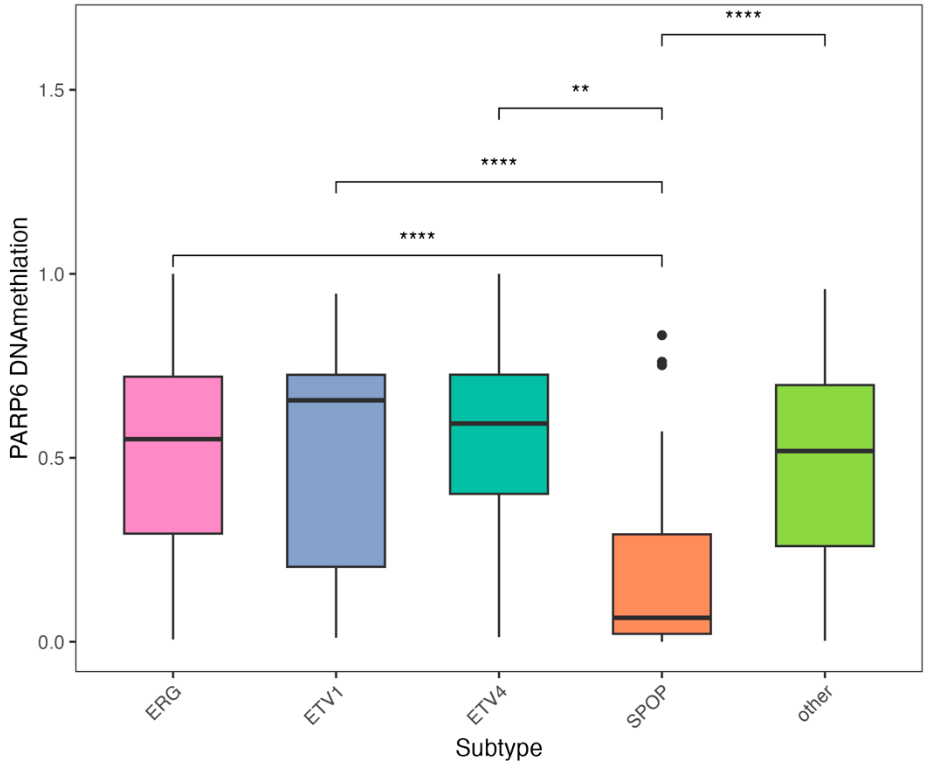

Figure S2. Bar chart of DNA methylation level of PARP6 in PRAD subtypes.

2. Independent dataset validation of MOTCS

We collected data from 1,272 breast cancer patients in the cBioPortal database. Since this dataset only has mRNA and DNA methylation datasets and lacks miRNA dataset, we could only conduct model validation based on single-omics data. The results are shown in Table S1.

Table S1. The external cohort validation

	ACC	F1_weighted	F1_macro
mRNA	0.344	0.240	0.157
DNAmethy	0.181	0.116	0.118

3. Model performance comparison of C in L1-SVC feature selection

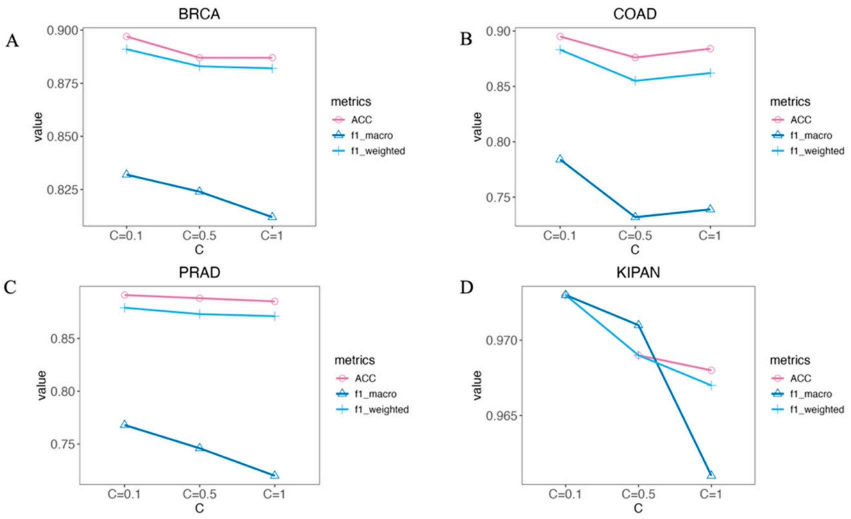

Figure S3. Line graph of model performance with different C values for different datasets. (A) BRCA, (B) COAD, (C) PRAD, (D) KIPAN.
